# Kinetic Analysis of CO_2_ Gasification for Maize Cob-Polypropylene Blended Biochar

**DOI:** 10.3390/ma19194070

**Published:** 2026-09-23

**Authors:** Ziming Wang, Yihe Cheng, Zhaofeng Guo, Long Ying, Jia-Hao Liu, Shao-Zheng Zhang, Xiaoke Yang

**Affiliations:** 1 Hebei Special Vehicle Modification Technology Innovation Center, Xingtai 054000, China; 2 Department of Automotive Engineering, Hebei Vocational University of Technology and Engineering, Xingtai 054000, China

**Keywords:** thermogravimetric analysis, biochar, CO_2_ gasification, kinetic

## Abstract

CO_2_ gasification of biomass represents a promising pathway toward carbon neutrality. While current research predominantly focuses on biomass carbonization with plastics or CO_2_ gasification of pure biochar, the critical relationship between physicochemical structures and gasification reactivity remains underexplored. The present work explores the CO_2_ gasification behavior of biochar derived from maize cob-polypropylene (PP) blends. The experimental results demonstrate that PP blending significantly enhances the gasification reactivity of maize cob-derived biochar. Structural characterization revealed that increasing the PP mass ratio led to an enhancement in specific surface area, which was the primary factor of biochar reactivity improvement. The thermogravimetric data were analyzed using both the random pore model (RPM) and unreacted core model (URCM). Comparative analysis confirmed that the RPM provided the best fit for the gasification process, with activation energy decreasing from 219.3 kJ/mol to 162.2 kJ/mol as the PP content increased from 0% to 60%.

## 1. Introduction

Fossil fuels, as a common fuel, generate a substantial amount of carbon dioxide during combustion, resulting in severe environmental damage [1,2]. The global emission of CO_2_ reached a total of 41.6 billion tons in 2024, an increase of 1 billion tons compared with that of 2023. Reducing carbon dioxide emissions and reducing dependency on fossil fuels present pressing global challenges. The carbon content generated by biochar is equal to the amount absorbed, making it a carbon-neutral resource [3]. Biochar allows for energy valorization of agricultural waste resources, reduces the dependence on fossil coal for blast furnace ironmaking and coal gasification, and addresses the growing scarcity of fossil fuels [4]. Biochar can be produced as a solid byproduct from the co-pyrolysis of plastic and biomass [3]. Prior to utilization, biomass resources undergo conversion through processes such as gasification, combustion, carbonization, and high-temperature pyrolysis. Gasification is a promising technology for biochar treatment. Pooya et al. explored the conversion of CO_2_ and carbon into CO as an energy fuel via gasification reactions [5]. These processes do not sequester CO_2_; instead, they are incorporated into the circulation system, providing fuel for various industrial applications. With the acceleration of the economy and technology, a large amount of plastic waste has led to severe ecological problems. For instance, polypropylene (PP) is widely used in the packaging industry, textile industry, medical equipment, and so on, making it one of the most widely produced plastics. Owing to the customizability of biochar to processing diverse biomass and plastic, the carbonization processes of biomass and plastics have garnered significant research interest. Esso et al. reported on the char-volatiles interactions during the carbonization processes of biomass components and PP [6]. They found that the char-volatile interactions altered the yield and the chemical structure of biochar. Qiang et al. demonstrated that PP addition improved the pore structure and specific surface area of biochar [7]. The carbonization of PP plastics and biomass enhances both the yield and carbon–hydrogen content of the resulting char product. Moreover, it results in a higher calorific value compared with that of biochar derived solely from lignocellulosic biomass [8,9,10]. While numerous research studies have delved into the carbonization processes for producing biochar from mixed biomass and plastic, the analyses of the impact of PP on the gasification processes of biochar are not sufficient.

In recent years, the influence of biochar structure on gasification reactions has attracted widespread attention. However, the structural characteristics influence gasification reactivity differently because of differences in the physicochemical properties of biochar. Duman et al. indicated that gasification of chars with higher surface area was found to be faster than that of chars having a lower surface area [11]. The studies demonstrate that the pore structure of char was the key factor influencing gasification. Wang et al. investigated the influence of alkali metals, such as potassium and sodium, on the structure evolution of coke and gasification reactivity via gaseous adsorption [12]. The results indicated that these alkali metals act as catalysts, thereby significantly improving the gasification reactivity of coke. Yun et al. demonstrated that decreasing the graphitization degree of biochar’s carbon structure promoted gasification reactivity [13]. In summary, there is no certain factor of microstructure for assessing the gasification reactivity of biochar derived from blends of maize cob (MC) and PP.

The CO_2_ gasification behavior of biochar has predominantly been analyzed in terms of its thermodynamic and kinetic characteristics. Wang et al. employed the unreacted core model (URCM), volume reaction model (VM), and random pore model (RPM) to calculate the kinetic parameters of petroleum coke and biomass char blends gasification in a CO_2_ atmosphere [14,15]. These results indicate that the RPM was more appropriate for describing the gasification reactivity curves. Everson et al. investigated the gasification of char behavior from eight kinds of coal using the URCM [16]. Their results demonstrated that RPM, compared with URCM, exhibited closer alignment with the experimental data. Zhang et al. employed a non-isothermal thermogravimetric approach to examine the CO_2_ gasification reactivity of distinct biomass components [17]. They observed that interactions among biomass components influenced tar composition and yield, resulting in increased production of CO during the Boudouard gasification process. Many studies have been performed on the gasification thermodynamics and kinetic characteristics of biochar and coke, but few studies have been performed on the gasification kinetics of biochar derived from blends of maize cob and PP.

While current research predominantly focuses on biomass carbonization with PP or CO_2_ gasification of pure biochar, the critical relationship between physicochemical structures and gasification reactivity remains underexplored. This study employed the non-isothermal method to investigate the gasification characteristics and kinetic behavior of biochar comprising PP and maize cob via a series of experiments. The kinetic parameters obtained provide a more accurate representation of the reaction process. Additionally, elemental and industrial analysis were conducted to determine the elemental composition of biochar. Scanning electron microscopy (SEM) and N_2_ adsorption were employed to analyze its pore structure. The mineral content of biochar was examined via X-ray diffraction (XRD), and its graphite microcrystalline structure was investigated via Raman spectroscopy. Employing RPM and URCM models, we investigated a kinetic gasification reaction process to explain the gasification reactivity of biochar, as well as the influence of temperature and other parameters on gasification reactivity. This study aims to enhance the reactivity of biochar during the gasification process and provide essential data for the conversion of CO_2_ into energy.

## 2. Materials and Methods

### 2.1. Sample Preparation

The samples used in this study included MC and PP materials. Maize cobs were collected from Dingzhou City, Hebei Province, China. Then, they were crushed using a crusher, sieved to a size smaller than 74 μm, and PP (China Sinopec, Beijing, China) a size smaller than 74 μm was selected. Proximate and ultimate analyses of the samples are shown in Table 1. The ash composition of MC carbon is detailed in Table 2. MC and PP blends were prepared in four different PP ratios (0, 20, 40, and 60 wt.%) and labeled as MC100, MC80PP20, MC60PP40, and MC40PP60, respectively. Samples with varying weight ratios were placed into a crucible and subjected to programmed thermal treatment in a controlled-atmosphere furnace under a N_2_ flow (200 mL/min) until a carbonization temperature of 973 K was reached, as illustrated in Figure 1.

### 2.2. Analysis of Physical and Chemical Properties

The microscopic morphology of the biochars was examined via SEM (Hitachi S4800, Tokyo, Japan) at 10 kV and 1000× magnification. The specific surface area and pore structure were determined employing a pore structure analyzer (Micromeritics TriStar II 3020, Micromeritics Instrument Corporation, Norcorss, GA, USA) with N_2_ adsorption at 77 K. The Brunauer–Emmett–Teller model was utilized to calculate specific surface areas. The mineral structures of the biochar were determined via X-ray powder diffractometry (PANalytical X’pert^3^, PANalytical B.V, Amalo, The Netherlands) using Cu Kα radiation (λ = 0.1541 nm) across a 10–90° scanning range. Raman spectroscopy was conducted utilizing a JY-HR800 laser confocal micro-Raman spectrometer (HORIBA LabRAM HR Evolution, Loos, France), employing a 2 mW laser with a wavelength of 532 nm as the excitation source. The measurement range for this analysis was 800–2000 cm^−1^.

### 2.3. Gasification Tests

Thermogravimetric analysis has proven effective in investigating the kinetics and mechanisms of the combustion and gasification processes of carbonaceous materials, such as metallurgical coke, petroleum coke, and biochar. This study conducted non-isothermal gasification tests employing a thermogravimetric analyzer (HENGJIU EXPERIMENTAL EQUIPMENT HCT-4, LTD, Beijing, China) in a CO_2_ atmosphere to determine the gasification behavior of the biochar samples. These samples were comminuted to particle sizes less than 74 μm. Samples approximately 5 mg in size were placed into an Al_2_O_3_ crucible (4 mm height × 5 mm diameter) for the non-isothermal gasification experiments. The samples were heated from an ambient temperature under a CO_2_ flow (100 mL/min) at heating rates of 5, 10, and 15 K/min until the final gasification temperature of 1473 K was reached. Each test was repeated three times to ensure the accuracy and reproducibility of the experiment. TG and DTG curves were automatically recorded using analysis software. The gasification conversion of the sample was calculated using the following equation:

(1) $$\alpha =\frac{m_{0}-m_{t}}{m_{0}-m_{\infty }}$$

where *m*_0_ represents the initial mass of the sample; *m_t_* is the mass of the sample at time t; and *m*_∞_ is the residual ash mass in the biochar following complete reaction.

### 2.4. Kinetic Analysis

Kinetic analysis provides essential information for gasification process design and optimization while enabling the quantitative characterization of sample properties.

The gasification of biochar materials involves a gas-solid heterogeneous reaction. The gasification reactivity (conversion rate) can be expressed using Equation (2):

(2) $$\frac{d\alpha }{dt}=kf\left(\alpha \right)$$

where *f (*α) represents the kinetic mechanism function in the gasification reaction, and k is the apparent reaction rate constant. The magnitude of k depends on the temperature and the Arrhenius equation in Equation (3):

(3) $$k=k_{0}exp\left(-\frac{E}{RT}\right)$$

where *k*_0_ is the pre-exponential factor, *E* is the activation energy, and *R* is the universal gas constant. Biochar gasification can be described by several kinetic models. In this study, the URCM and the Random Pore Model were employed to study biochar reactivity, both of which are widely accepted approaches in non-isothermal kinetic analysis of biomass char [12,18,19,20]. Among these models, the URCM model assumes that porous particles consist of homogeneous nonporous spherical grains and the reaction initially occurs on the external surface of the sample and gradually progresses inward [21]. However, the RPM model accounts for the overlap of pore indications and reduces the reaction area [22,23]. Both these models were applied to study the gasification performance of the samples. The gasification rates of RPM and URCM are represented by Equations (4) and (5), respectively.

(4) $$\frac{d\alpha }{dt}=k_{RPM}{exp}^{\frac{-E}{RT}}\left(1-\alpha \right)\sqrt{1-\psi ln\left(1-\alpha \right)}$$

(5) $$\frac{d\alpha }{dt}=k_{URCM}{exp}^{\frac{-E}{RT}}{\left(1-\alpha \right)}^{\frac{2}{3}}$$

(6) $$\psi =\frac{4\pi L_{0}(1-\varepsilon_{0})}{S_{0}^{2}}$$

In Equation (4), ψ is the parameter describing the particle structure, *L*_0_ is the pore length, and *S*_0_ is the pore surface area.

In non-isothermal experiments, the temperature *T* at time *t* is expressed by Equation (7):

(7) $$T=T_{0}+\beta \cdot t$$

where *T*_0_ is the initial reaction temperature and *β* is the heating rate. The kinetic parameters were evaluated under non-isothermal conditions employing a constant heating rate. To facilitate analysis, the explicit time dependence in Equation (5) was removed by applying the standard transformation:

(8) $$\frac{d\alpha }{dT}=\frac{k_{URCM}}{\beta }e^{-E/RT}{\left(1-\alpha \right)}^{\frac{2}{3}}\;$$

Equation (8) can be integrated to yield:

(9) $$\alpha =1-{\left(1-\frac{k_{URCM}E}{3\beta R}p(u)\right)}^{3}$$

where

(10) $$u=\frac{E}{RT}$$

The approximate solutions have been proposed in the literature [15,24] and are expressed as:

(11) $$p(u)=\frac{e^{-u}}{u^{2}}$$

Consequently, Equation (9) can be written as:

(12) $$\alpha =1-{\left\{1-\frac{k_{0}RT^{2}}{3\beta E}exp\left(\frac{-E}{RT}\right)\right\}}^{3}$$

Similarly, Equation (4) can be integrated to yield:

(13) $$\alpha =1-exp\left\{-\left[k_{0}\frac{RT^{2}}{\beta E}exp\left(\frac{-E}{RT}\right)\right]\left[1+\frac{k_{0}\psi }{4}\frac{RT^{2}}{\beta E}exp\left(\frac{-E}{RT}\right)\right]\right\}$$

By fitting the experimental results, the parameters in the RPM and URCM dynamic models, including *E*, *k*_0_, and *ψ*, can be obtained.

## 3. Results and Discussion

### 3.1. Fundamental Characteristics of Samples

#### 3.1.1. Porous Structure Analysis

Gil et al. investigated the correlation between gasification reactivity and raw material characteristics [25]. Prior studies have indicated that gasification reactivity is significantly influenced by ash content, volatile matter content, particle size, specific surface area, and pore morphology. In this study, SEM was employed to observe the differences in pore structure among the samples.

Figure 2 presents the SEM images of the particle shapes and surface structures of the four biochars. Each sample consisted of irregularly shaped, approximately elliptical particles exhibiting surface protrusions. Notably, the biochars were composed of irregular elliptical particles, exhibiting irregular protrusions on the surface and no prominent open pores; their structures primarily comprised small and micro pores. SEM analysis revealed that differences in surface structures were linked to varying PP contents. As the PP content increased, the structures exhibited a substantial number of cracks and pores. Furthermore, the microporous structures determined the specific surface area of the samples, making it difficult to obtain accurate conclusions from SEM images. To accurately characterize the structure of different samples, N_2_ adsorption experiments must be conducted to study the specific surface area and pore structure distribution of the samples.

Table 3 lists the pore-structure characteristics of different samples. The average pore size of the samples decreases as the PP content increases. The total pore volume of MC40PP60 was 0.0156 cm^3^/g, and that of MC100 was 0.0117 cm^3^/g. The sample MC40PP60 exhibited the highest specific surface area and total pore volume. The specific surface area of MC40PP60 was 17.71 m^2^/g, which is approximately three times greater than that of MC100 (6.63 m^2^/g). Consequently, MC40PP60 exhibits a more developed pore structure and an increased specific surface area. Wu et al. demonstrated that the final pyrolysis temperature influences the pore structure of carbon and that a higher final pyrolysis temperature promotes a secondary reaction between volatiles and carbon, thereby generating abundant pore structures [26]. These pores have been observed to readily interconnect and expand during the gasification reaction, which contributes to the high reactivity of biochars. Furthermore, Chun et al. demonstrated that the gasification reactivity increased as the specific surface area increased [27]. The optimum gasification reactivity was observed in MC40PP60.

#### 3.1.2. X-Ray Diffraction Analysis

Gong et al. demonstrated that the microcrystalline structure of char plays a critical role in gasification reactivity and that mineral content also influences the development of the microcrystalline structure [28]. To further analyze the influence of minerals on the biochar structure, XRD was conducted on the biochar samples.

Figure 3 presents the XRD patterns of the samples. The minor peaks observed in the figure are attributed to inorganic minerals in the biomass. Wang et al. demonstrated that alkali metal vapors disrupt the original aromatic ring structure of coke during adsorption [12]. The alkali metal content decreased as the PP content increased. Consequently, MC40PP60 exhibited a more ordered carbon structure. The sharp peak that appears at approximately 27° is attributed to SiO_2_. Aladejebi et al. demonstrated that SiO_2_ hinders the gasification reactivity of coke analogs in CO_2_ [29]. As the PP content increased, the intensity of the SiO_2_ peak gradually decreased, indicating that the inhibitory effect of SiO_2_ on the biochar gradually decreased. Furthermore, the (002) peak at approximately 23° corresponds to a graphite-like microcrystalline structure, indicating the presence of a small amount of layered graphite structure. The intensity of the (002) peak exhibited only slight variation with increasing PP content. This indicates that the minerals in the biomass may moderately influence the graphitic microcrystalline structure of the sample.

#### 3.1.3. Raman Spectroscopy Analysis

Raman spectroscopy was used to further investigate the impact of varying PP contents on the carbon microcrystalline structure of the samples. The Raman spectra (800–1800 cm^−1^) shown in Figure 4 represent the samples comprising different PP contents.

The observed Raman spectra primarily reflect the differences in the carbon structures of biochars with varying PP contents, characterized by two prominent peaks at approximately 1350 cm^−1^ and 1580 cm^−1^, which correspond to the D peak (reflecting disorder or defects in the carbon structure) and G peak (reflecting the number of layers and integrity of the carbon structure lattice) [30]. Quantitative analysis of the changes in the carbon structure of different samples is challenging based solely on the D and G peaks owing to the significant overlap between the D and G peaks, which complicates the correlation of the Raman spectra with specific structural parameters [31]. To address this, the orientation parameters of the overlapping peaks were derived via spectral fitting. Sadezky et al. observed that the Raman spectra can be categorized into five distinct peaks: D_1_, D_2_, D_3_, D_4_, and G [31]. The D_1_ peak typically represents impurity atoms, sp^2^ hybridized carbon atoms, or in-plane defects within the basic structural units. The G_1_ peak is typically related to the stretching vibration mode with E_2g_ symmetry in graphitic microcrystalline structures, which is related to all the paired sp^2^ crystal carbon atoms in the graphitic network. Following Wang et al., who reported that Lorentzian and Gaussian functions are more suitable for fitting Raman spectra [12], we applied these functions in this study for improved data fitting.

As can be seen in Table 4, MC60PP40 samples exhibit higher D_1_ and D_2_ band intensities compared to MC40PP60, indicating a high degree of graphitization at MC40PP60. Conversely, the G band intensity is higher in MC60PP40 than in MC40PP60, suggesting greater graphitization in MC60PP40. The previous analysis showed that it was difficult to judge the degree of graphitization of the biochar based on the intensity of peaks D_1_, D_2_, D_3_, D_4,_ and G. According to Sheng et al., the ratios I_D1_/I_G_ and I_G_/I_ALL_ provide a more reliable representation of the graphitization degree [32]. Thus, the band area ratios I_D_/I_G_ and I_G_/I_ALL_ were utilized to reflect the degree of graphitization of biochar. As shown in Table 4, as the PP content increases, the I_D1_/I_G_ values decrease and the I_G_/I_ALL_ values increase. The data suggest a slight increase in the orderliness of the carbon structure, with a trend of MC40PP60 > MC60PP40 > MC80PP20 > MC100.

### 3.2. Thermogravimetric Analysis of MC-PP Biochar

The gasification reactivity of the biochar composed of PP and MC was tested at three different heating rates, as shown in Figure 5. The gasification behavior was characterized quantitatively by calculating some crucial parameters such as the initial gasification reaction temperature (*T_i_*), the peak reaction rate temperature (*T_m_*), and the end temperature of the gasification reaction (*T_f_*) [33]. The final results are shown in Table 5.

It is evident that the order of the T_i_ values for the four samples is MC40PP60 < MC60PP40 < MC80PP20 < MC100. The gasification reaction temperature (*T_i_*) of MC40PP60 was 1041 K, which was 1096 K for MC100. The gasification curves at different heating rates demonstrated that as the heating rate increased, the curves moved toward the high-temperature zone. It was found that as the heating rate increased, the *T_i_*, *T_f_* and *T_m_* increased relatively. Regardless of the heating rate, MC40PP60 (*T_i_*) was lower than MC100. Meanwhile, the peak reaction rate temperature (*T_m_*) of MC40PP60 was higher than that of MC100, and the endpoint temperature (*T_f_*) of MC40PP60 gasification reaction was lower than that of MC100. From these phenomena indications, the reactivity of MC40PP60 was obviously higher than that of MC100. The proportion of PP significantly influences the gasification reaction of biochar-PP. Figure 6 shows the TG curves for the PP and MC blends obtained at a heating rate of 15 K/min. The mixed gasification reaction of MC and PP did not satisfy the additivity principle; therefore, the gasification reaction process of the blend was influenced by the interactions between the two samples. As the PP content increased, the TG curve gradually shifted toward the low-temperature region, indicating an improvement in the gasification reactivity of the biochar. The final trend in gasification reactivity was MC40PP60 > MC60PP40 > MC80PP20 > MC100; PP did not contain alkali metals. It can be seen from Table 2 that the total content of K2O and Na_2_O in ash of MC100 is 51.01%. According to Wang et al. [18], K_2_O and Na_2_O exhibit catalytic effects on the gasification reaction. However, Since MC biomass contains a relatively low ash content, the catalytic effect of inherent minerals on the gasification reaction is expected to be minor.

Raman spectroscopy analysis revealed the most ordered carbon structure at MC40PP60, indicating that the carbon structure was not the dominant factor influencing gasification reactivity. Furthermore, N_2_ adsorption demonstrated that MC40PP60 exhibited the highest specific surface area, indicating that the influence of pore structure on biochar was the key factor.

### 3.3. Kinetic Parameter

In the previous study [12,15], the kinetic parameters, including E, *K_0_*, and ψ, were obtained based on the fitted gasification data at four heating rates. The kinetic parameters of different samples were investigated using the RPM and URCM models by employing a nonlinear least-square fitting method. Following the RPM and URCM models described in Section 2.4, the kinetic parameters (*E*, *K_0_*, and R^2^) were calculated by fitting the gasification reaction data collected at the three different heating rates. Figure 7 shows the calculation of the dx/dt curves utilizing the kinetic parameters from Equations (4) and (5) at three different heating rates. The RPM model successfully predicted the maximum reaction rate during the reaction process by considering the pore overlap effect. Initially, the growth of cylindrical pores increased the total reaction surface area, thereby increasing conversion. Eventually, pore coalescence between adjacent pores occurs, reducing the reactive surface area and consequently diminishing the reaction rate. In contrast, the URCM model failed to describe the maximum reaction rate but predicted that the reaction rate would continue to decline throughout the process. Figure 8 presents the fitting results of the various models under varying heating rates, and the correlation coefficients (R^2^) are listed in Table 6.

The fitting parameters were incorporated into Equations (8) and (9), which were subsequently used to predict the biochar gasification process to further study the accuracy of these two models. The data are presented in Table 6, and both models provide reliable predictions. The R^2^ value for the RPM model approaches 1 more closely than that for the URCM model, indicating that the RPM model describes the mechanism of the reaction kinetics process with greater precision. Gao et al. demonstrated that RPM exhibited better characterization performance than URCM for porous materials such as biochar [34]. Similarly, Zhang et al. investigated the gasification of biomass char and reached similar conclusions [35]. Furthermore, biochar was used as a porous material by analyzing its physical and chemical characteristics. According to the RPM model, the gasification activation energy of MC100 was 219.3 kJ/mol. The activation energies are close to that of the published activation energies for biochar [15,36]. As the PP content increased, the activation energy of the biochar gradually decreased. As shown in Table 6, both the activation energy (*E*) and pre-exponential factor (*K*) decreased. This phenomenon is referred to as the dynamic compensation effect. Wang et al. observed this phenomenon when studying the gasification reaction of biomass [15].

## 4. Conclusions

This study investigated the gasification characteristics and kinetic behavior of biochar derived from a mixture of PP and MC. As the PP content increased, the TG curve gradually shifed toward the low-temperature region, indicating an improvement in the gasification reactivity of the biochar. Structural characterization revealed that increasing the PP mass ratio led to an enhancement in specific surface area, which was the primary factor of biochar reactivity improvement. The kinetic fitting results indicated that the RPM model accurately described char co-gasification with CO_2_ compared with the URCM model. The activation energy calculated using the RPM model ranged from 162.2 to 219.3 kJ/mol. The activation energy decreased with the increase of PP mass ratio.

## Figures and Tables

**Figure 1:**
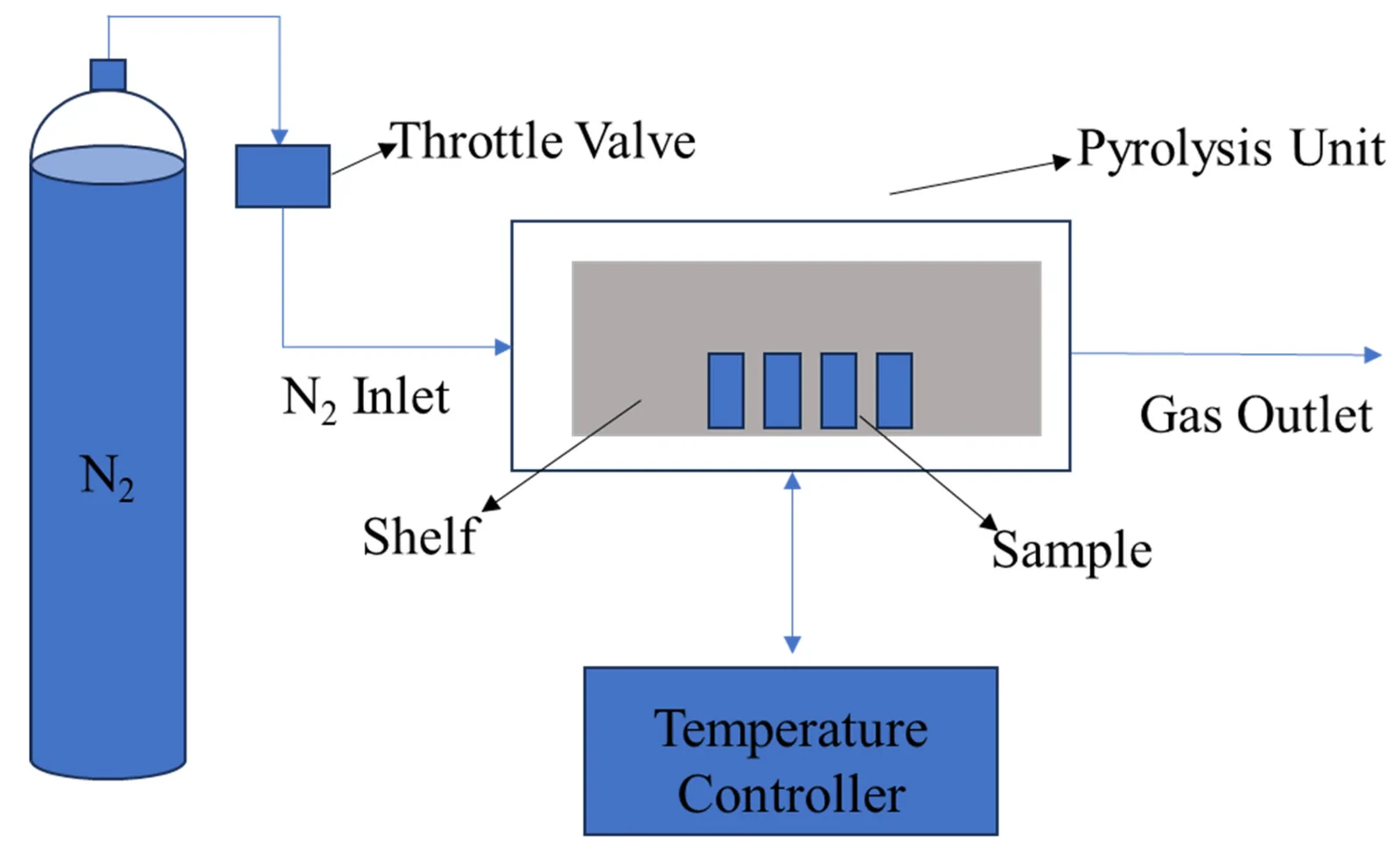
Schematic diagram of the high-temperature pyrolysis unit.

**Figure 2:**
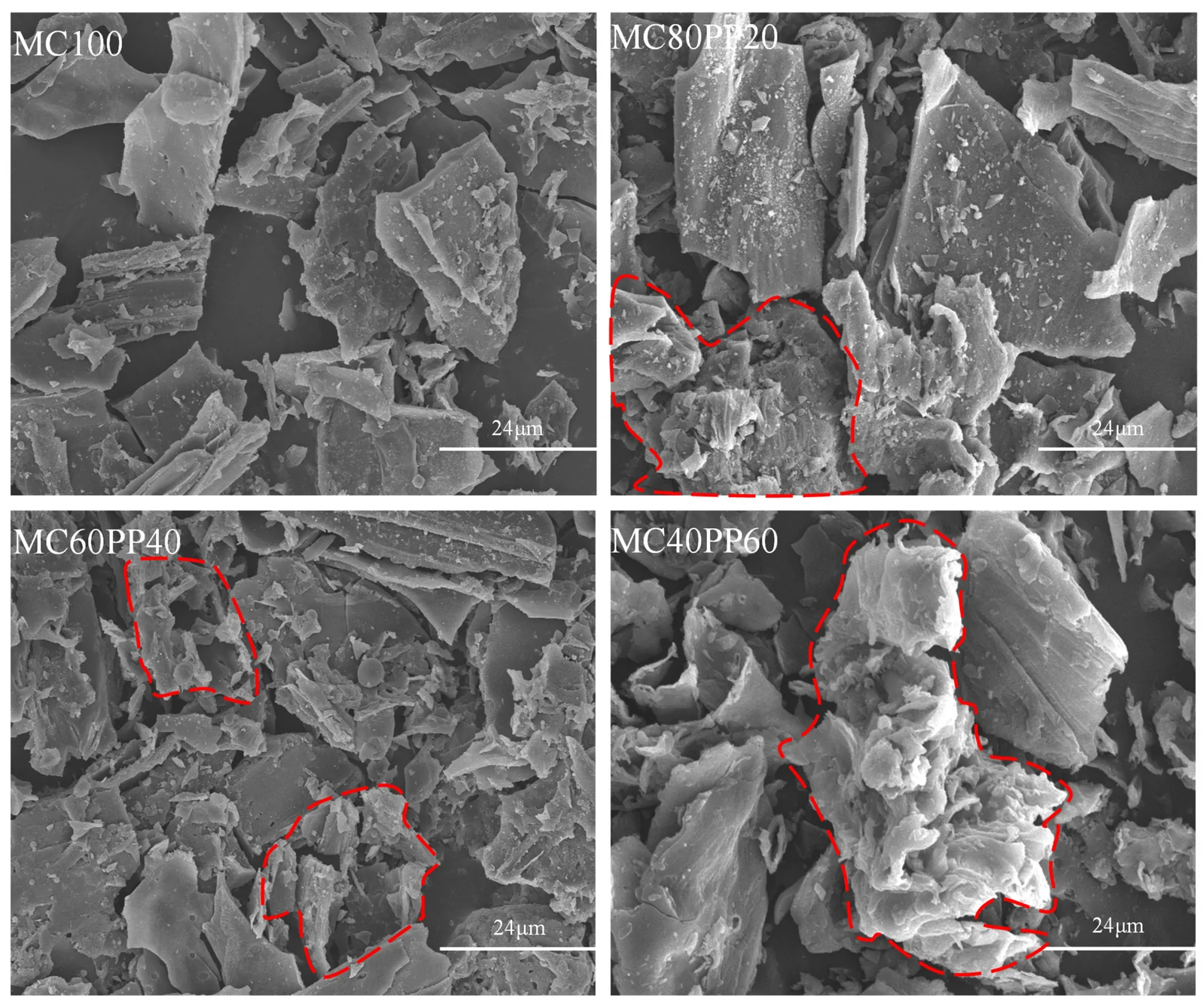
The SEM photographs result of different MC-PP biochar.

**Figure 3:**
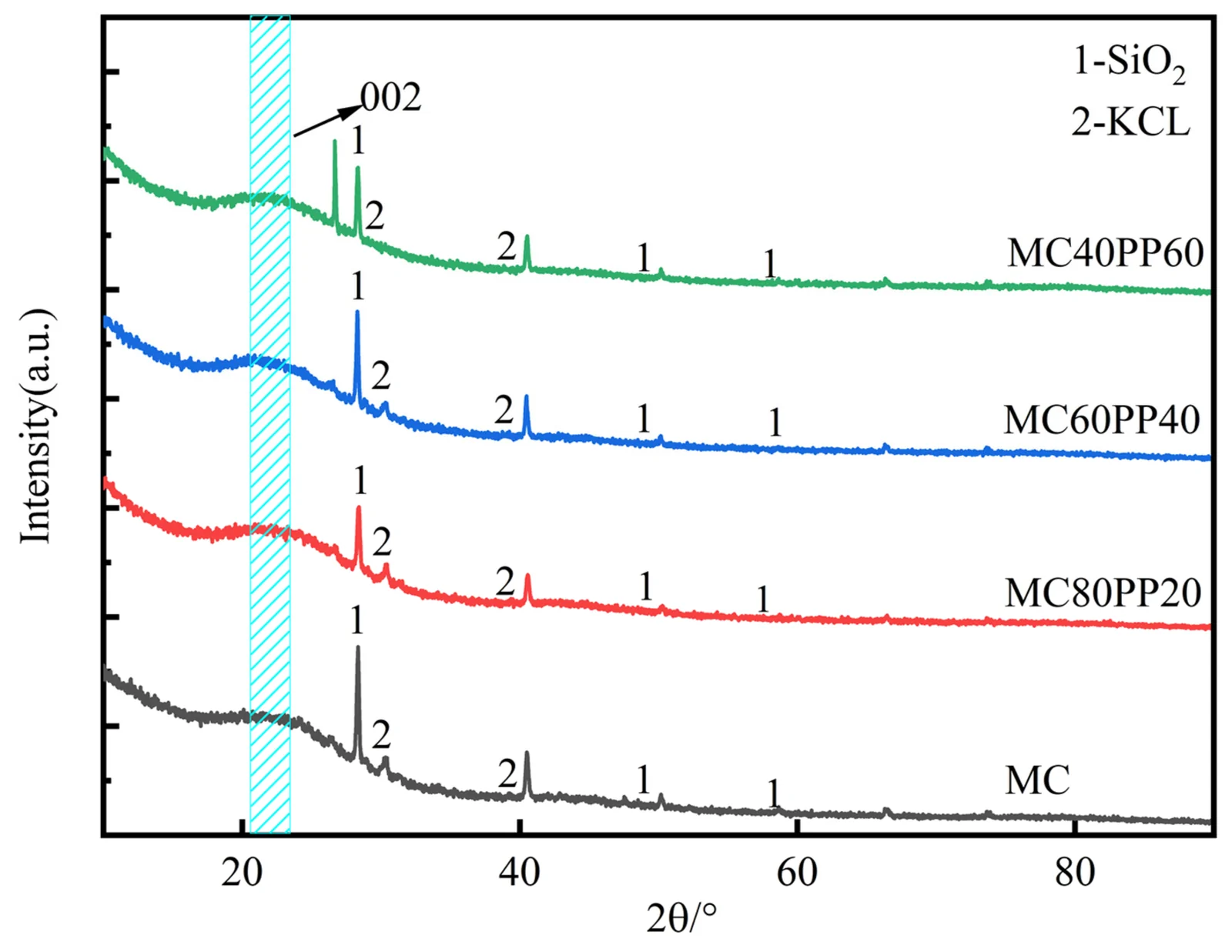
XRD patterns of different biochars.

**Figure 4:**
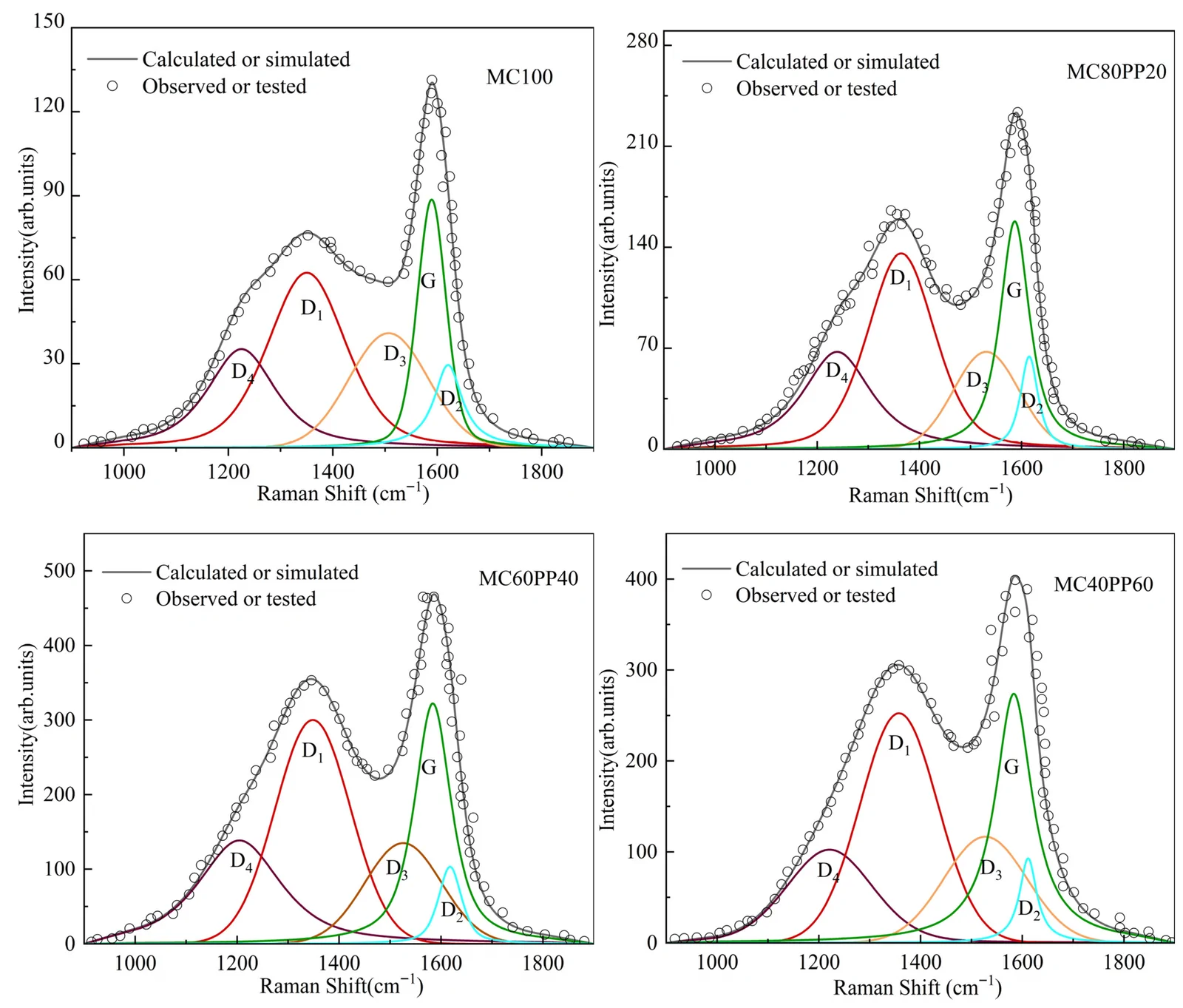
Raman spectra deconvolution of the different biochar of MC-PP.

**Figure 5:**
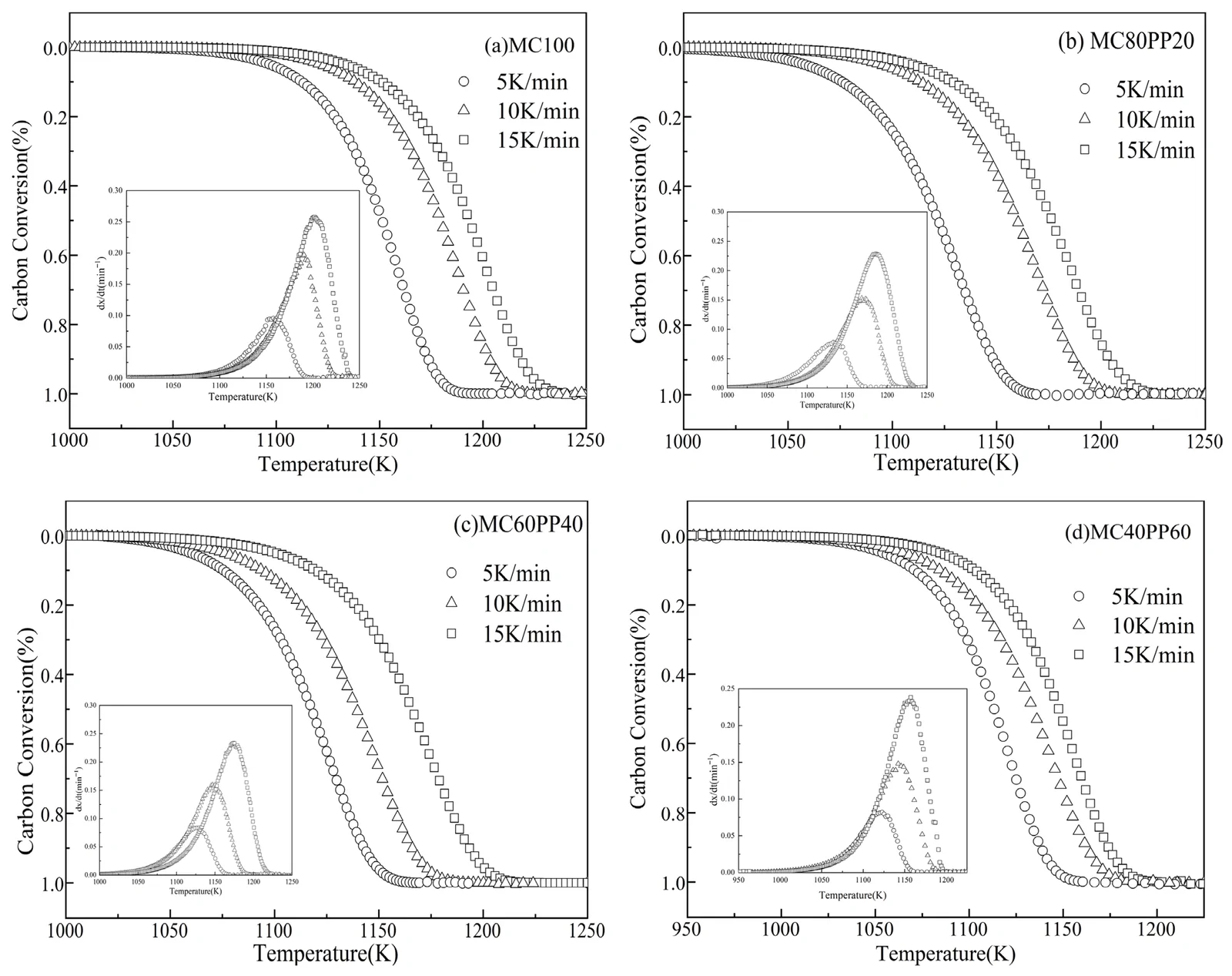
TG and DTG curves of gasification for the MC-PP biochar at heating rates of 5, 10, and 15 K/min.

**Figure 6:**
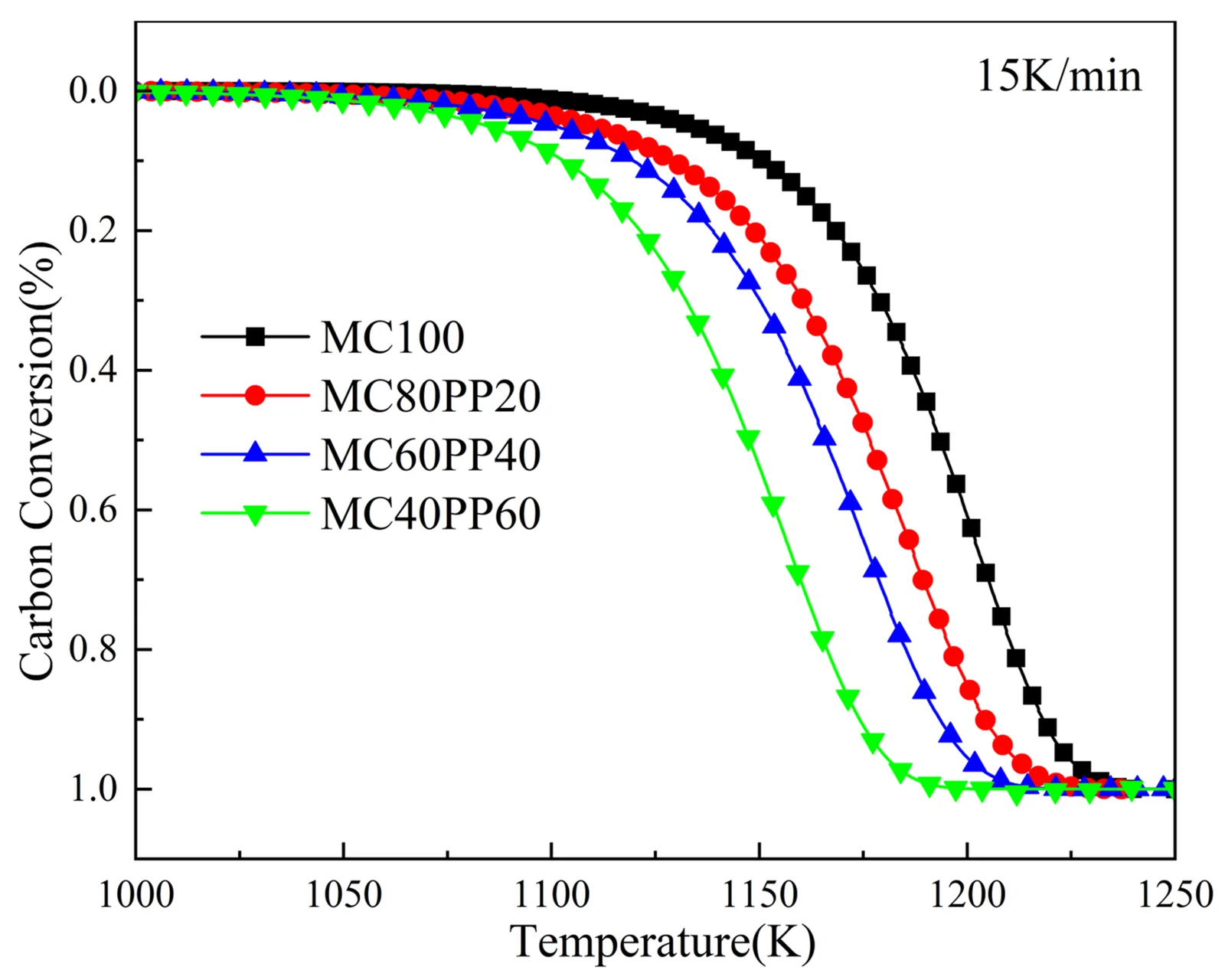
Gasification curves of the biochars at a heating rate of 15K/min.

**Figure 7:**
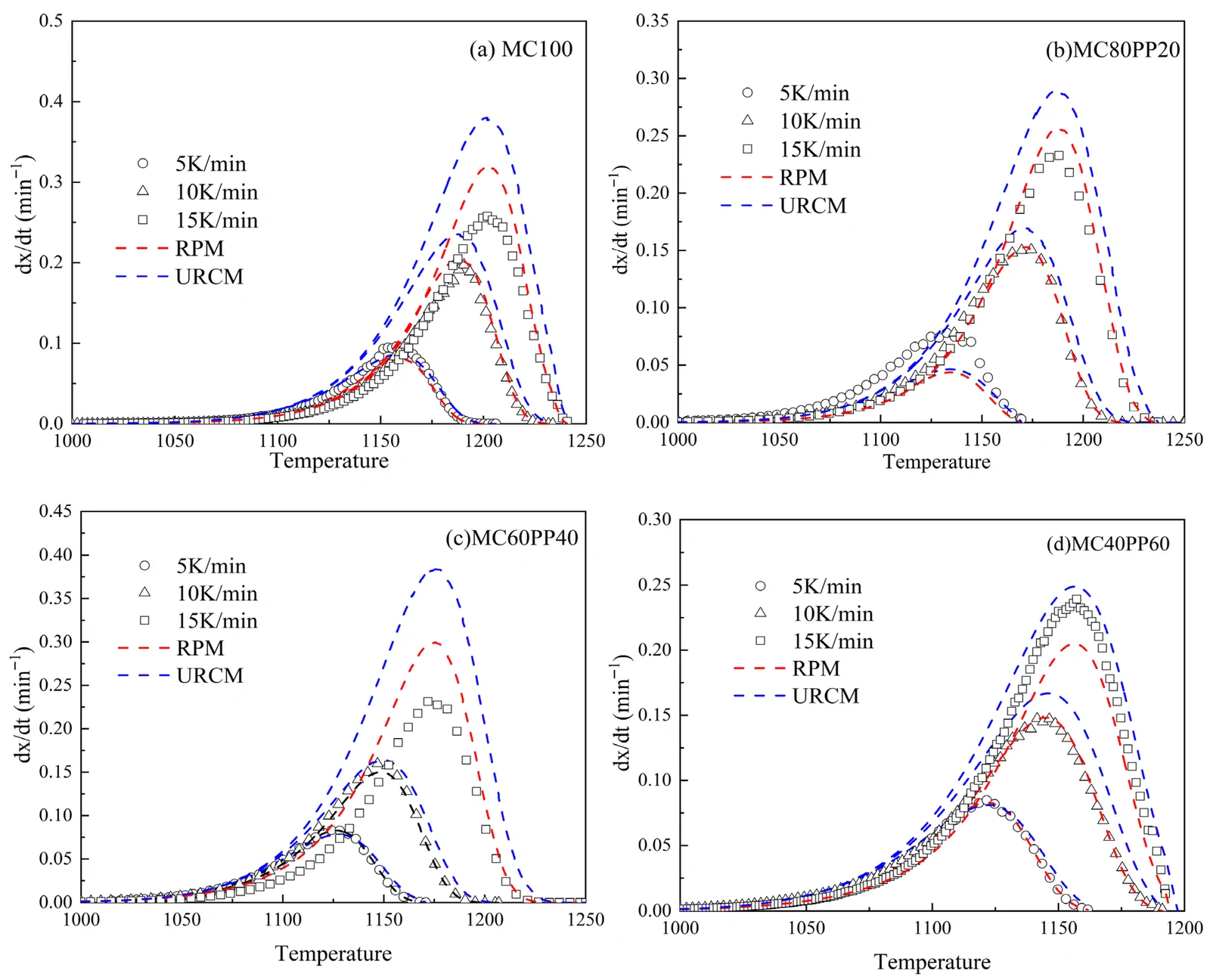
Reaction rate curves of different biochars and two model fitting curves.

**Figure 8:**
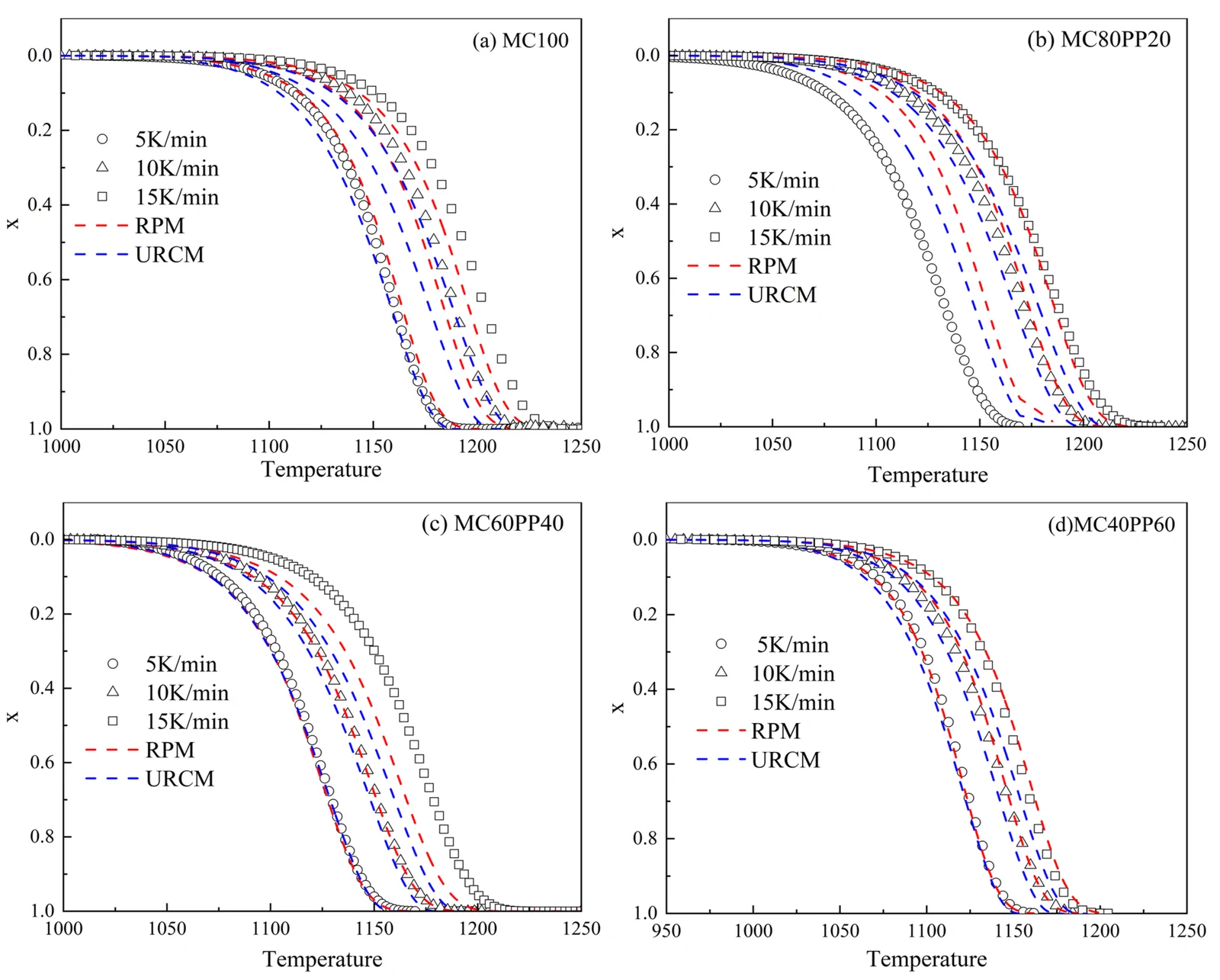
Comparison of experimental values and fitting results of conversion rate.

**Table 1:** Proximate analysis and ultimate analyses of MC and PP.

Samples	Proximate Analysis (wt.%)			Ultimate Analysis (wt.%)				
	FC_d_	A_d_	V_d_	C_d_	H_d_	N_d_	S_d_	O_d_
MC	16.3	1.65	82.42	46.56	4.90	0.42	0.34	45.62
PP	0.04	0.04	99.92	83.55	13.99	0.08	0.01	2.39

Note: FC: fixed carbon, A: ash, V: volatile matter, C: carbon, H: hydrogen, N: nitrogen, S: sulfur, O: oxygen.

**Table 2:** Ash compositions of the MC-char.

Sample	SiO_2_	Al_2_O_3_	Fe_2_O_3_	CaO	Na_2_O	K_2_O	MgO
MC-char	25.73	1.96	2.39	3.76	0.56	50.45	0.82

**Table 3:** Pore characteristic parameters of the co-pyrolysis biochar of MC-PP.

Sample	S_BET_	TPV	APW
MC100	6.63	0.0117	8.42
MC80PP20	7.05	0.0104	6.32
MC60PP40	8.86	0.0096	4.81
MC40PP60	17.71	0.0156	4.20

Note: S_BET_: total surface area, m^2^/g; TPV: total pore volume, m^3^/g; APW: average pore diameter, nm.

**Table 4:** Characteristic parameters of Raman spectra in the different biochars of MC-PP.

Sample	I_D1_	I_D2_	I_D3_	I_D4_	I_G_	I_D1_/I_G_	I_(D3+D4)_/I_G_	I_G_/I_ALL_
MC100	11,938	2389	7742	5621	6575	1.815	2.032	0.191
MC80PP20	23,160	3435	10,852	11,195	13,519	1.713	1.630	0.217
MC60PP40	55,488	6688	25,363	23,370	32,742	1.694	1.488	0.227
MC40PP60	48,196	5220	23,658	19,854	29,572	1.629	1.471	0.233

**Table 5:** Gasification characteristic parameters of MC-PP biochar at a heating rate of 15 K/min.

Samples	*T_i_*/K	*T_m_*/K	*T_f_*/K
MC	1096	1201	1240
MC80PP20	1066	1185	1237
MC60PP40	1057	1175	1232
MC40PP60	1041	1157	1204

**Table 6:** Kinetic parameters of different MC-PP biochars for two kinetic models.

Samples	RPM			URCM		
	*E* (kJ/mol)	*K*_0_ (min^−1^)	R^2^	*E* (kJ/mol)	*K*_0_ (min^−1^)	R^2^
MC100	219.3	7.04472 × 10^11^	0.9982	236.0	1.41125 × 10^13^	0.9975
MC80PP20	212.1	3.90634 × 10^11^	0.9975	218.6	1.86047 × 10^12^	0.9962
MC60PP40	168.5	1.52253 × 10^9^	0.9988	208.4	8.98465 × 10^11^	0.9971
MC40PP60	162.2	5.55621 × 10^8^	0.9989	201.0	3.80442 × 10^11^	0.9973

## Data Availability

The original contributions presented in this study are included in the article. Further inquiries can be directed to the corresponding author.

## References

[1] Holechek J.L., Geli H.M., Sawalhah M.N., Valdez R. (2022). A global assessment: Can renewable energy replace fossil fuels by 2050?. Sustainability.

[2] Wang J., Azam W. (2024). Natural resource scarcity, fossil fuel energy consumption, and total greenhouse gas emissions in top emitting countries. Geosci. Front..

[3] Adeniyi A.G., Iwuozor K.O., Emenike E.C., Ajala O.J., Ogunniyi S., Muritala K.B. (2024). Thermochemical co-conversion of biomass-plastic waste to biochar: A review. Green Chem. Eng..

[4] Emenike E.C., Adeleke J., Iwuozor K.O., Ogunniyi S., Adeyanju C.A., Amusa V.T., Okoro H.K., Adeniyi A.G. (2022). Adsorption of crude oil from aqueous solution: A review. J. Water Process. Eng..

[5] Lahijani P., Zainal Z.A., Mohammadi M., Mohamed A.R. (2015). Conversion of the greenhouse gas CO_2_ to the fuel gas CO via the Boudouard reaction: A review. Renew. Sustain. Energy Rev..

[6] Esso S.B.E., Yan Q., Xiong Z., Hu X., Xu J., Jiang L., Su S., Hu S., Wang Y., Xiang J. (2022). Importance of char-volatiles interactions during co-pyrolysis of polypropylene and biomass components. J. Environ. Chem. Eng..

[7] Hu Q., Zhang H., Mao Q., Zhu J., Zhang S., Yang H., Chen H. (2024). The effect of co-pyrolysis of bamboo waste and polypropylene on biomass deoxygenation and carbonization processes. Energy.

[8] Adeniyi A., Ighalo J., Onifade D., Popoola A. (2020). Production of hybrid biochar by retort-heating of elephant grass (*Pennisetum purpureum*) and low density polyethylene (LDPE) for waste management and product development. J. Mater. Env. Sci..

[9] Jerzak W., Bieniek A., Magdziarz A. (2023). Multifaceted analysis of products from the intermediate co-pyrolysis of biomass with Tetra Pak waste. Int. J. Hydrog. Energy.

[10] Song B., Hall P. (2020). Densification of biomass and waste plastic blends as a solid fuel: Hazards, advantages, and perspectives. Front. Energy Res..

[11] Duman G., Uddin M.A., Yanik J. (2014). The effect of char properties on gasification reactivity. Fuel Process. Technol..

[12] Wang G., Ren S., Zhang J., Ning X., Liang W., Zhang N., Wang C. (2020). Influence mechanism of alkali metals on CO_2_ gasification properties of metallurgical coke. Chem. Eng. J..

[13] Yun C., An M., Ban Y., Zhou H., Hao J., Li N., Zhi K., Wang Y., Liu Q. (2025). Insight into the structural properties and CO_2_ gasification reactivity of biomass and lignite co-pyrolysis char. Fuel.

[14] Gupta A., Thengane S.K., Mahajani S. (2018). CO_2_ gasification of char from lignocellulosic garden waste: Experimental and kinetic study. Bioresour. Technol..

[15] Wang G., Zhang J., Zhang G., Ning X., Li X., Liu Z., Guo J. (2017). Experimental and kinetic studies on co-gasification of petroleum coke and biomass char blends. Energy.

[16] Everson R.C., Neomagus H.W., Kaitano R., Falcon R., Cann V.M.D. (2008). Properties of high ash coal-char particles derived from inertinite-rich coal: II. Gasification kinetics with carbon dioxide. Fuel.

[17] Zhang S., Wu M., Bie X., Qian Z., Li Q., Zhang Y., Zhou H. (2024). Deciphering interactions between biomass components during CO_2_ gasification: Insights from thermogravimetric behavior, gas production, and char reactivity. Fuel.

[18] Wang G., Zhang J., Huang X., Liang X., Ning X., Li R. (2018). Co-gasification of petroleum coke-biomass blended char with steam at temperatures of 1173–1373 K. Appl. Therm. Eng..

[19] Alsawadi A.M., Marsh R., Steer J.M., Morgan D. (2025). A study of the mechanisms associated with CO2 utilisation via the reverse Boudouard reaction. Fuel.

[20] Ye L., Zhang J., Xu R., Ning X., Zhang N., Wang C., Mao X., Li J., Wang G., Wang C. (2022). Co-combustion kinetic analysis of biomass hydrochar and anthracite in blast furnace injection. Fuel.

[21] Ochoa J., Cassanello M., Bonelli P., Cukierman A. (2001). CO_2_ gasification of Argentinean coal chars: A kinetic characterization. Fuel Process. Technol..

[22] Bhatia S.K., Perlmutter D. (1981). A random pore model for fluid-solid reactions: II. Diffusion and transport effects. AIChE J..

[23] Bhatia S.K., Perlmutter D. (1980). A random pore model for fluid-solid reactions: I. Isothermal, kinetic control. AIChE J..

[24] Leroy V., Cancellieri D., Leoni E., Rossi J.-L. (2010). Kinetic study of forest fuels by TGA: Model-free kinetic approach for the prediction of phenomena. Thermochim. Acta.

[25] Gil M.V., Riaza J., Álvarez L., Pevida C., Rubiera F. (2015). Biomass devolatilization at high temperature under N_2_ and CO_2_: Char morphology and reactivity. Energy.

[26] Wu L., Guan Y., Zhou J., Jiang X., Liu T., Pan J. (2023). Effects of variable amounts of volatiles in corncob on microwave co-pyrolysis of low-rank coal and corncob. Fuel.

[27] Chun D.D., Ni D., Simson A. (2022). The effect of inherent inorganics and CO_2_ co-pyrolysis on biochar production from biowastes and their gasification reactivity. Biomass Bioenergy.

[28] Gong X., Guo Z., Wang Z. (2009). Variation of char structure during anthracite pyrolysis catalyzed by Fe_2_O_3_ and its influence on char combustion reactivity. Energy Fuels.

[29] Aladejebi O.A. (2017). Effect of Minerals on Coke Analogue Carbon Bonding and Reactivity. Ph.D. Thesis.

[30] Beyssac O., Goffé B., Petitet J.-P., Froigneux E., Moreau M., Rouzaud J.-N. (2003). On the characterization of disordered and heterogeneous carbonaceous materials by Raman spectroscopy. Spectrochim. Acta Part A Mol. Biomol. Spectrosc..

[31] Sadezky A., Muckenhuber H., Grothe H., Niessner R., Pöschl U. (2005). Raman microspectroscopy of soot and related carbonaceous materials: Spectral analysis and structural information. Carbon.

[32] Sheng C. (2007). Char structure characterised by Raman spectroscopy and its correlations with combustion reactivity. Fuel.

[33] Xu R.-S., Zhang J.-L., Wang G.-W., Zuo H.-B., Zhang P.-C., Shao J.-G. (2016). Gasification behaviors and kinetic study on biomass chars in CO_2_ condition. Chem. Eng. Res. Des..

[34] Gao P., Liu Y., Huang X., Abulaiti A., Yang S. (2023). Effect of wet torrefaction on the physicochemical characteristics and gasification behavior of biochar. Ind. Crops Prod..

[35] Matsumoto K., Takeno K., Ichinose T., Ogi T., Nakanishi M. (2009). Gasification reaction kinetics on biomass char obtained as a by-product of gasification in an entrained-flow gasifier with steam and oxygen at 900–1000 °C. Fuel.

[36] Xiao R., Yang W. (2016). Kinetics characteristics of straw semi-char gasification with carbon dioxide. Bioresour. Technol..

